# Prevalence, morphological variation and ossification of sesamoid bones of the forefoot: a retrospective radiographic study of 8,716 Chinese subjects

**Published:** 2016-08-15

**Authors:** Tao Sun, Lingxiang Wang, Haitao Zhao, Wenjuan Wu, Wenhai Hu

**Affiliations:** 1 *Department of Orthopedic Surgery, Third Hospital of Hebei Medical University, Shijiazhuang* 050051, *Hebei Province, China*; 2 *Department of Gynecology, Forth Hospital of Hebei Medical University, Shijiazhuang* 050011, *Hebei Province, China*; 3 *Department of Radiology, Third Hospital of Hebei Medical University, Shijiazhuang* 050051, *Hebei Province, China*

**Keywords:** sesamoid bones, metatarsophalangeal joint, prevalence, ossification, radiological

## Abstract

**Background and Aim:** Previous studies provided evidence of a genetic basis for the occurrence of sesamoids bone in the foot among different ethnic populations. However, information for the Chinese population has not been previously reported. Therefore, the aim of our study was to determine the distribution, morphological variation and ossification timeline of sesamoid bones of the forefoot in a large sample of the Chinese population.

**Methods:** Antero-posterior (AP) and oblique radiographs of 4,417 left and 4,299 right feet obtained from 8,716 patients in the Hebei province of Northern China, and retrospectively examined for the presence of sesamoid bones, identified as a small oval bone plantar to each metatarsophalangeal (MTP) joint and the first interphalangeal (IP) joint.

**Results and Conclusions:** The prevalence rate of a sesamoid bone associated with the first to fifth MTP joints and the first IP joint was 99.96%, 3.08%, 0.39%, 0.69%, 8.94%, and 59.22%, respectively. Moreover, a morphological variation in hallucal sesamoids was identified in 12.09% of feet, with variations classified into three distinct types according to bone size and the number of ossification centers. Ossification begins in the hallucal MTP and IP joints at approximately 8 years of age, with the final ossification center being evidence for the sesamoid bone of the fourth MTP joint at 28 years of age.

**Relevance for patients:** Our study provides important anatomical data regarding the prevalence of sesamoid bones in the forefoot of a large population of Chinese adult and pediatric patients for use in clinical practice and research in forensic science and anthropology.

## 1. Introduction

Sesamoid bones are round or oval-shaped small bones, which vary significantly in terms of occurrence, shape and distribution. In fact, the occurrence rate of sesamoid bones varies widely, from < 1% to approximately 100%, with some sesamoid bones always being present, such as the patella and the hallucal sesamoids [1], while others occur rarely, such as the os talotibiale (0.5%) and os intercuneiforme (0.03%) [2]. The hallucal sesamoids have been well studied due to their consistent presence in humans and their relatively common implication in pathologies of the foot, including fractures, infections, arthritis, and osteonecrosis [3,4]. By contrast, there is little information available regarding the occurrence rate of the sesamoids of the second to fifth toe, with the prevalence of these sesamoid bones known to vary greatly among different ethnic groups [3]. The Han Chinese population is the world’s largest single ethnic group, constituting over 18.8% of the global population in 2011 [5]. A specific evaluation of the occurrence of sesamoid bones of the forefoot has yet to be conducted with a Chinese population.

With regard to the developmental timeline of the sesamoid bones of the forefoot, only one study has evaluated the ossification age of hallucal sesamoid bones in children [6]. In their study, Dharap et al. reported enchondral ossification, initiated in chondrification centers, appeared only at 8 years of age in girls, with a later onset in boys [6]. It has been proposed that absent, hypoplastic or multi partite hallucal sesamoid bones result from absent or incomplete ossification or from the fusion of multiple ossification centers [7-9]. Morphological variations of hallucal sesamoid bones are classified based on the size of the sesamoids and the number of ossification centers visible.

The aim of our study was to determine the prevalence of sesamoid bones in the forefoot and the morphological variations of hallucal sesamoids in a large group of Chinese individuals from the Hebei province in Northern China, as well as to identify the age of onset of ossification of these sesamoid bones, through a retrospective analysis of foot radiographs.

## 2. Participants and methods

Foot radiographs used in our analysis were obtained from patients referred to the Trauma Centre of the Orthopedic Hospital in the Hebei province, between November 2005 and September 2012. Our methods were approved by our hospital’s Institutional Review Board. Due to the retrospective nature of our study, the need for informed consent was waived.

Radiographic images were retrieved using the Picture Archiving and Communication System. Patient records, and all other information obtained from the system, were anonymized and re-identified prior to analysis. High-quality anterior-posterior (AP) and oblique radiographs, with all metatarsal and phalangeal bones of the feet, for patients ≥ 5 years of age, were used in the analysis. Radiographic images for patients with forefoot pathologies, such as amputated toes and tumors, were excluded. Overall, 8,716 radiographic images were included in our analysis, with 8,207 images provided by 5,157 men and 3,050 women (4,163 images of the left foot and 4,044 of the right), with an additional 337 provided by boys and 172 by girls, 5 to 17 years old. Radiographs from pediatric patients were used to determine the onset and completion of ossification of the hallucal sesamoid bones.

Per protocol, the presence of a sesamoid bone had to be independently confirmed by all members of the research team, which consisted of the orthopedic chief surgeon at our hospital, an attending surgeon and a radiologist. Presence of sesamoid bones in the forefoot was confirmed by identification of a small oval bone plantar to the MTP joint or IP joint of the hallux or toes. A consensus between all three members of the team was necessary prior to calculating the prevalence. The type of sesamoid bone was classified based on the size of the bone and the number of ossification centers visible on standard AP and oblique images.

Collected data included the patient’s sex, age, foot laterality (right/left), location, number of sesamoid bones, and type of sesamoid bone. All data were recorded in spreadsheet for analysis (Microsoft Excel 2007™).

## 3. Results

### 3.1. Prevalence of sesamoids at the MTP and IP joints of the hallux

**Figure 1:**
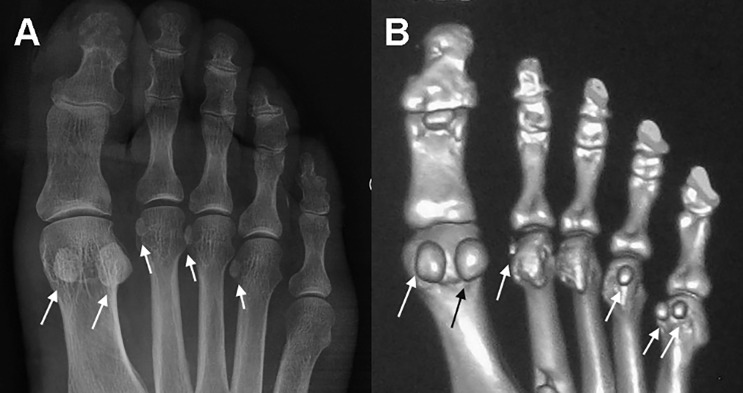
Anteroposteriorradiographs (A) and three dimensional reconstruction computed tomography scans (B) of the forefoot of two adults, with the location of sesamoid bones at the metatarsophalangeal joints indicated by arrows.

**Figure 2:**
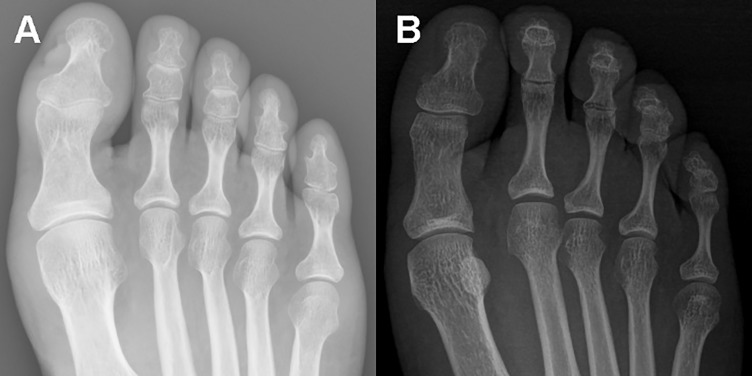
Anteroposterior radiograph of the forefoot in two adults: (A) absence of both hallucal sesamoid bones, which is a rare variation; (B) absence of the medial sesamoid bone at the hallucal metatarsophalangeal joint.

The radiographic appearance and coexistence of sesamoid bones in the forefoot are shown for representative cases in Figure 1, with the distribution and incidence rate for adults, 18 to 92 years of age, summarized in Table 1. Sesamoid bones plantar to the hallucal MTP joint were identified in 8,204 radiographs, for a prevalence rate of 99.96%. Among these images, paired sesamoid bones were identified on 8,086 radiographs, with a single lateral sesamoid bone identified on118 radiographs, for a prevalence rate of 98.53% for paired and 1.44% for single hallucal sesamoid bones. An absence of hallucal sesamoid bones was identified on only 3 (0.04%) radiographs (Figure 2). None of the 8,207 radiographs showed a single medial hallucal sesamoid bone. However, a single sesamoid bone was identified plantar to the IP joint of the hallux in 4,860 (59.22%) of radiographs.

### 3.2. Prevalence of sesamoids at the second, third, fourth, and fifth toe

The prevalence rate of sesamoid bones at the MTP joint of the second, third, fourth, and fifth toe were 3.08%, 0.39%, 0.69%, and 8.94%, respectively. A single sesamoid bone and paired sesamoid bones were identified, respectively, in 249 (3.03%) and 4 (0.05%) radiographs at the second MTP joints, in 50 (0.61%) and 7 (0.09%) radiographs at the fourth MTP joints, and in 521 (6.35%) and 213 (2.60%) radiographs at the fifth MTP joints. No cases were identified with two sesamoid bones at the MTP joint of the third toe. Coexistence of sesamoid bones at two or more MTP joints and the hallucal IP joint was identified (Figure 1).

**Table 1:** Distribution and incidence rate of sesamoid bones in the forefoot of adults (*n* = 8,207)

			Left Feet					Right Feet		
Sex	MTP: (Hallux)	MTP: (2nd toe)	MTP: (3rd toe)	MTP: (4th toe)	MTP: (5th toe)	IP: (Hallux)	MTP: (Hallux)	MTP: (2nd toe)	MTP: (3rd toe)	MTP: (4th toe)	MTP: (5th toe)	IP: (Hallux)
Male	2586	66	10	21	243	1365	2569	75	8	14	232	1465
Female	1575	54	10	10	135	1046	1474	58	4	12	124	984
Total	4,161 (99.95)	120 (2.88)	20 (0.48)	31 (0.74)	378 (9.08)	2,411 (57.91)	4,043 (99.98)	133 (3.29)	12 (0.30)	26 (0.64)	356 (8.80)	2,449 (60.56)

Notes: MTP, metatarsophalangeal; IP, interphalangeal.

### 3.3 Prevalence of morphological variations in sesamoids at the hallucal MTP joint

**Figure 3:**
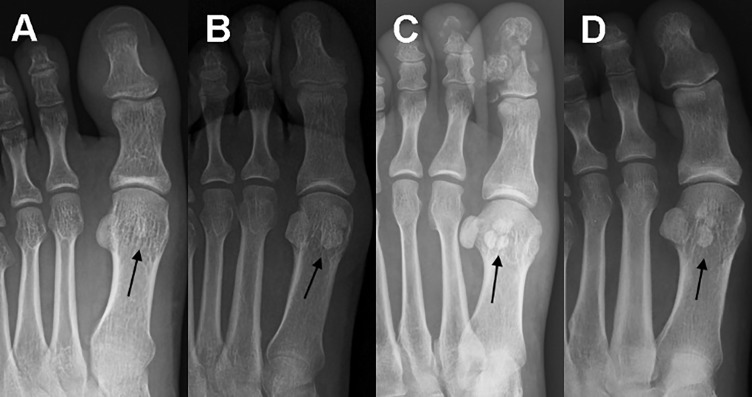
Anteroposterior radiographs of the forefoot of three adults, showing morphological variation among sesamoid bones, identified with the arrow (black): (A) Type I, one ossification center and hypoplastic sesamoid; (B) Type II, two ossification centers; and (C and D) Type III, ≥ three ossification centers.

On the majority of images, both hallucal sesamoids were elliptical in shape, the lateral being slightly smaller than the medial. Morphological variation of the sesamoid bones plantar to the hallucal MTP joint was identified on 992 (12.09%) radiographs. The variation in hallucal sesamoids can be classified into three distinct types (Figure 3) based on the size and the number of ossification centers visible on standard AP and oblique views on plain radiographs (Table 2). The three types of morphological variation are defined as follows: type I, one ossification center with a hypoplastic bone; type II, two ossification centers; and type III, ≥ three ossification centers. The distribution of morphological variations in our dataset of 992 radiograph with sesamoid bones plantar to the MTP joint was as follows: type I, 43.75%; type II,55.14%; and type III,1.11%.

**Table 2:** Classification of sesamoid bones at the hallux metatarsophalangeal joint

Type	Description	Medial Sesamoid *n* (%)	Lateral Sesamoid *n* (%)	Bilateral Sesamoid *n* (%)
I	One ossification center and hypoplastic sesamoid	428 (5.22)	6 (0.07)	---
II	Two ossification centers	508 (6.19)	22 (0.27)	17 (0.21)
III	≥ 3 ossification centers	10 (0.12)	---	1 (0.01)

### 3.4. Onset of ossification of the sesamoid bones of the forefoot

**Figure 4:**
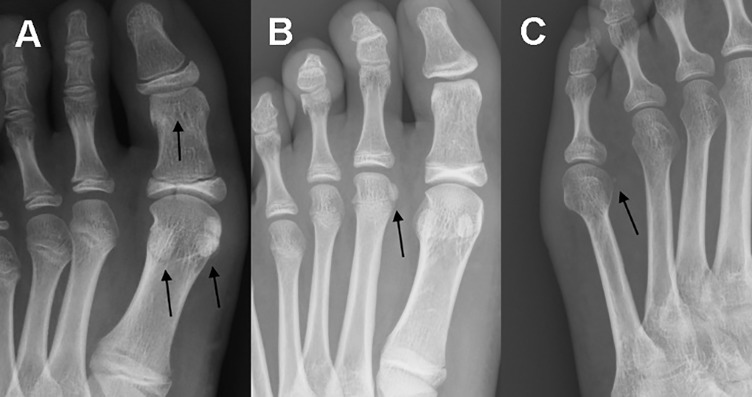
Anteroposterior radiographs of the forefoot of three children, showing the onset appearance of sesamoid bones at the: (A) hallucal metatarsophalangeal and interphalangeal joints; (B) second metatarsophalangeal joint; and (C) fifth metatarsophalangeal joint. Radiographs were obtained from an 8 year old girl (A), a 12 year old boy (B) and a 12 year old girl (C).

The onset of ossification of the sesamoid bones of the forefoot is reported in Table 3, with an example of the first presence of sesamoid bones in the forming skeletal structure of children shown in Figure 4. Generally, the first ossification center for the hallucal sesamoids appeared at 8 years of age, and at 28 years of age for the sesamoid bone plantar to the fourth MTP joint. The age of completion of ossification of sesamoid bones at the hallucal MTP joint and IP joint, based on the 509 radiographs of pediatric patients, 5 to 17 years old, is reported in Table 4. Ossification of the hallucal sesamoids was completed by 12 years in girls and 13 years in boys.

**Table 3:** Age of onset of ossification of the sesamoid bones in the forefoot

Sites	Males; age (years)	Females; age (years)
MTP: (Hallux)	8	8
MTP: (2nd toe)	12	10
MTP: (3rd toe)	21	27
MTP: (4th toe)	20	28
MTP:(5th toe)	15	12
IP: (Hallux)	10	8

Notes: MTP, metatarsophalangeal; IP, interphalangeal.

**Table 4:** Presence/absence of ossification of the hallucal sesamoid bones in boys and girls (5–17 years)

	Boys		Girls	
Age, years (Count)	Present	Absent	Present	Absent
5 (*n* = 24)	–	15	–	9
6 (*n* = 24)	–	17	–	7
7 (*n* = 27)	–	19	–	8
8 (*n* = 24)	1	16	3	4
9 (*n* = 27)	5	6	12	4
10 (*n* = 33)	14	4	12	3
11 (*n* = 41)	9	7	23	2
12 (*n* = 48)	25	8	15	–
13 (*n* = 36)	21	–	15	–
14 (*n* = 43)	31	–	12	–
15 (*n* = 41)	31	–	10	–
16 (*n* = 66)	50	–	16	–
17 (*n* = 75)	58	–	17	–

## 4. Discussion and Conclusion

### 4.1. Prevalence of sesamoids at the hallucal MTP and IP joints

An overall prevalence of hallucal sesamoid bones of 99.96% was identified in our large study group representative of the Chinese population, a prevalence rate that is comparable to previously reported data for other populations (Table 5) [1,6,10-12]. Although most researchers consider the presence of two sesamoids at the hallucal MTP joint to be consistent in humans, a number of anatomical variations of the hallucal sesamoid have been described, including: paired sesamoids, absent medial or lateral, and complete absence [3,7,13-15]. We identified 11 cases of morphological variation of the hallucal sesamoid bones in the literature, 6 cases of an absent medial sesamoid; 4 cases of an absent lateral (fibular) sesamoid; and 4 cases of complete absence [3,16]. The frequency distribution of the morphological variants of the hallucal sesamoid bone has not been systematically reported. Based on the large sample size of our dataset, including 8207 radiographs of adult feet, we calculated that paired sesamoids were the most common type, with a prevalence rate of 98.53%, with a single lateral sesamoid identified in 118 radiographs (1.44%) and an absence sesamoid identified in three radiographs (0.04%), with no identification of a single hallucal sesamoid. However, previous studies reported an incidence rate of hallucal sesamoid bones of 100% in European, British, Malawian, Bahrainis, and Turkish, while only one article reported an incidence rate of congenital absence of 1.47% in adults, which may be associated with chronic sesamoiditis, painful bipartite sesamoid bones and chronic non-union fractures [17].

Morphological variations of the hallucal sesamoids identified in our study included hypoplastic and multi-partite variations. A hypoplastic sesamoid was more common in medial, than lateral, hallucal sesamoids. Partite sesamoids are clinically meaningful as they may cause pain and are very difficult to differentiate from a sesamoid bone fracture [8,18]. Several radiographic ‘hints’ are helpful in making a differential diagnosis between a partite and fracture of the hallucal sesamoid bone. Namely, a fracture usually appears as an irregular serrated line, with sharp pointed corners and an irregular cleavage plane, whereas partite sesamoids are usually larger than their uni-partite counterpart [19]. The frequency of partition of hallucal sesamoids varied widely, ranging from 3.3% to 19% [6,19], with a higher prevalence in feet with a hallux valgus deformity, compared to those with a normal hallux alignment [20]. In our study, we identified a prevalence rate of partite hallucal sesamoids of 6.8%, which is in the medium-to-low range of previously reported results. Similar to hypoplastic sesamoids, partite sesamoids were more commonly identified in the medial hallucal sesamoid, and occasionally in the lateral or paired hallucal sesamoids.

The highest prevalence rate of sesamoids was at the MTP joint of the hallux, followed by the IP joint of the hallux. However, the true prevalence of a sesamoid bone at the hallucal IP joint is somewhat controversial, due to significant differences in identification of this sesamoid bone between anatomical (cadaveric) and radiological evaluation. Anatomical studies have reported a prevalence rate of sesamoid bones at the hallucal IP joint of 72.5% to 93.8%, compared to the prevalence rate of 50.6% to 56.3% reported in radiographic studies [6,12,15,21,22]. We identified a prevalence rate of a sesamoid bone at the hallucal IP joint of 59.22%, which was comparable to the rate reported by Masaki [15]. Differences in reported prevalence rates may result from differences in radiographic methods. In fact, Masaki demonstrated that the identification rate of a sesamoid bone at the hallucal IP joint could increase by 56.3% to 93.8% when using a 1/4 sensitivity intensifying screen [15]. According to anatomical studies, a single sesamoid at the hallucal IP joint is present in 52.5% of adults, with a paired sesamoid identified in a further 20% of adults for an overall prevalence rate of 72.5% [21]. We did not identify a case of two sesamoid bones at the hallucal IP joint, and identified only one reported case of this rare finding in the literature [23].

**Table 5:** Incidence rate and distribution of sesamoid bones: comparisonto previously published data

Site	Pfitzner^a^(1892)	Bizarro (1921) [20]	Msamati & Igbigbi (2001) [10]	Dharap et al. (2007) [6]	Coskun et al. (2009) [1]	Sun et al. (present study)
MTP (Hallux)	100%	100%	100%	100%	100%	99.96%
MTP: (2nd toe)	1.6%	1%	–	2.1%	0.4%	3.08%
MTP: (3rd toe)	–	1%	–	0.6%	0.2%	0.39%
MTP: (4th toe)	–	2%	–	0.6%	0.1%	0.69%
MTP: (5th toe)	5.5–6.2%	10%	10.8%	12.1%	4.3%	8.94%
IP: (Hallux)	50.6%	5%	–	3.9%	2%	59.22%
Population	European	British	Malawian	Bahrainis	Turkish	Chinese
No. of feet	385	100	126	336	984	8,207

Notes: MTP, metatarsophalangeal joint; IP, interphalangeal joint;

a adapted from Bizarro (1921) and Yammine (2014).

The prevalence rate of sesamoid bones at the second through fifth metatarsals in our dataset varied between 0.39% and 8.94%. Therefore, these sesamoid bones were not as consistently present as the hallucal sesamoids. Previous studies have reported the presence of only one sesamoid bone at these locations as being typical. Dharap et al. [6] reported presence of two sesamoids in the fifth toe. We also identified a few cases of two sesamoids at the second, fourth and fifth toe, a new finding not previously reported. No cases of two sesamoids were identified at the third toe. Among these occurrences of two sesamoid bones in our dataset, 8.94% of cases were located at the fifth toe. This prevalence rate is higher than the rate of 4.3% to 6.2% reported by Coskun et al. [1] and Pfitzner [24], but lower than the 10% to 12.1% reported by Bizarro [12], Dharap et al. [6], and Msamati and Igbigbi [10].

In our subset of radiographs from pediatric patients, we identified that the ossification of sesamoids in the forefoot progresses gradually over the first three decades of life. Sesamoid bones of the hallucal MTP and IP joints begin to ossify during the first decade of life, those of the second and fifth MTP joints during the teenage years, and those of the third and fourth MTP joints in the third decade of life. Previous research has reported the ossification of the hallucal sesamoid bones to begin at approximately 8 years in females and 9 to 11 years in males, with ossification completed by 10 to 12 years of age [6,22]. In the same way that ossification begins 1-2 years earlier in girls than in boys, epiphyseal union of the sesamoid bones is achieved 1-2 years earlier in the second decade of life in females than in males [25]. This knowledge regarding the timeline of ossification and epiphyseal union in sesamoid bones could assist us in estimating the age of unidentified skeletal more accurately. The timeline of ossification of the sesamoid bones at the other MTP and IP has not been previously reported. Although identifying the appearance of the first ossification center of sesamoid bones is relatively easy from plain radiographs, identifying the end-point of ossification of sesamoid bones is more difficult. Therefore, it is possible that the end-point of ossification identified in our study may only represent the early part of epiphyseal union and, therefore, of the overall timeline of ossification. We recommend that the end-point of ossification be defined as the lower of the two adjacent ages between which there is no significant difference in the identification of a sesamoid bone.

The limitations of our study need to be acknowledged in the interpretation of our results. Foremost, our results are limited by the retrospective design of our study, which included only Chinese individuals who presented for diagnosis and treatment of a foot injury at our hospital. Moreover, computed tomography and magnetic resonance imaging may be more sensitive methods to identify non-ossified sesamoid bones. However, plain radiography is the routine examination recommended for evaluation of foot injuries in our hospital, and is the imaging technique of choice in most orthopedic departments. Despite these limitations, our sample size of 8,716 images is the largest dataset currently available and provides a robust representative dataset of the prevalence rate of sesamoid bones of the forefoot in the Chinese population.

In conclusion, our comprehensive study of sesamoid bones in representative group of the adult and pediatric Chinese populations is the first to characterize the distribution and prevalence rate of sesamoid bones at each MTP and IP joints. We are also the first to have provided a timeline of ossification of sesamoid bones of the forefoot, showing a progression from ossification of the hallucal sesamoids in the first decade to ossification of the remaining sesamoids extending to the third decade of life. Our comprehensive dataset will be of value to clinical practice, as well as to researchers in forensic science and anthropology.
